# Bilinearity in Spatiotemporal Integration of Synaptic Inputs

**DOI:** 10.1371/journal.pcbi.1004014

**Published:** 2014-12-18

**Authors:** Songting Li, Nan Liu, Xiao-hui Zhang, Douglas Zhou, David Cai

**Affiliations:** 1 Department of Mathematics, MOE-LSC and Institute of Natural Sciences, Shanghai Jiao Tong University, Shanghai, China; 2 State Key Laboratory of Cognitive Neuroscience and Learning and IDG/McGovern Institute for Brain Research, Beijing Normal University, Beijing, China; 3 Courant Institute of Mathematical Sciences and Center for Neural Science, New York University, New York, New York, United States of America; 4 NYUAD Institute, New York University Abu Dhabi, Abu Dhabi, United Arab Emirates; The Krasnow Institute for Advanced Studies, United States of America

## Abstract

Neurons process information via integration of synaptic inputs from dendrites. Many experimental results demonstrate dendritic integration could be highly nonlinear, yet few theoretical analyses have been performed to obtain a precise quantitative characterization analytically. Based on asymptotic analysis of a two-compartment passive cable model, given a pair of time-dependent synaptic conductance inputs, we derive a bilinear spatiotemporal dendritic integration rule. The summed somatic potential can be well approximated by the linear summation of the two postsynaptic potentials elicited separately, plus a third additional bilinear term proportional to their product with a proportionality coefficient 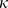. The rule is valid for a pair of synaptic inputs of all types, including excitation-inhibition, excitation-excitation, and inhibition-inhibition. In addition, the rule is valid during the whole dendritic integration process for a pair of synaptic inputs with arbitrary input time differences and input locations. The coefficient 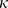 is demonstrated to be nearly independent of the input strengths but is dependent on input times and input locations. This rule is then verified through simulation of a realistic pyramidal neuron model and in electrophysiological experiments of rat hippocampal CA1 neurons. The rule is further generalized to describe the spatiotemporal dendritic integration of multiple excitatory and inhibitory synaptic inputs. The integration of multiple inputs can be decomposed into the sum of all possible pairwise integration, where each paired integration obeys the bilinear rule. This decomposition leads to a graph representation of dendritic integration, which can be viewed as functionally sparse.

## Author Summary

A neuron, as a fundamental unit of brain computation, exhibits extraordinary computational power in processing input signals from neighboring neurons. It usually integrates thousands of synaptic inputs from its dendrites to achieve information processing. This process is known as dendritic integration. To elucidate information coding, it is important to investigate quantitative spatiotemporal dendritic integration rules. However, there has yet to be extensive experimental investigations to quantitatively describe dendritic integration. Meanwhile, most theoretical neuron models considering time-dependent synaptic inputs are difficult to solve analytically, thus impossible to be used to quantify dendritic integration. In this work, we develop a mathematical method to analytically solve a two-compartment neuron model with time-dependent synaptic inputs. Using these solutions, we derive a quantitative rule to capture the dendritic integration of all types, including excitation-inhibition, excitation-excitation, inhibition-inhibition, and multiple excitatory and inhibitory inputs. We then validate our dendritic integration rule through both realistic neuron modeling and electrophysiological experiments. We conclude that the general spatiotemporal dendritic integration structure can be well characterized by our dendritic integration rule. We finally demonstrate that the rule leads to a graph representation of dendritic integration that exhibits functionally sparse properties.

## Introduction

For information processing, a neuron receives and integrates thousands of synaptic inputs from its dendrites and then induces the change of its membrane potential at the soma. This process is usually known as dendritic integration [1]–[3]. The dendritic integration of synaptic inputs is crucial for neuronal computation [2]–[4]. For example, the integration of excitatory and inhibitory inputs has been found to enhance motion detection [5], regularize spiking patterns [6], and achieve optimal information coding [7] in many sensory systems. They have also been suggested to be able to fine tune information processing within the brain, such as the modulation of frequency [8] and the improvement of the robustness [9] of gamma oscillations. In order to understand how information is processed in neuronal networks in the brain, it is important to understand the computational rules that govern the dendritic integration of synaptic inputs.

Dendritic integration has been brought into focus with active experimental investigations (see reviews [1], [10] and references therein). There have also been many theoretical developments based on physiologically realistic neuron models [11], [12]. Among those works, only a few investigate quantitative dendritic integration rules for a pair of excitatory and inhibitory inputs [3], [13] and there has yet to be an extensive investigation of the integration of a pair of excitatory inputs or a pair of inhibitory inputs. In this work, we propose a precise quantitative rule to characterize the dendritic integration for all types of synaptic inputs and validate this rule via realistic neuron modeling and electrophysiological experiments.

We first develop a theoretical approach to quantitatively characterize the spatiotemporal dendritic integration. Initially, we introduce an idealized two-compartment passive cable model to understand the mathematical structure of the dendritic integration rule. We then verify the rule by taking into account the complicated dendritic geometry and active ion channels. For time-dependent synaptic conductance inputs, we develop an asymptotic approach to analytically solve the cable model. In this approach, the membrane potential is represented by an asymptotic expansion with respect to the input strengths. Consequently, a hierarchy of cable-type equations with different orders can be derived from the cable model. These equations can be analytically solved order by order using the Green's function method. The asymptotic solution to the second order approximation is shown to be in excellent agreement with the numerical solutions of the original cable model with physiologically realistic parameters.

Based on our asymptotic approach, we obtain a new theoretical result, namely, a nonlinear spatiotemporal dendritic integration rule for a pair of synaptic inputs: the summed somatic potential (SSP) 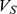 can be well approximated by the summation of the two postsynaptic potentials 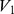 and 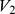 elicited separately, plus an additional third nonlinear term proportional to their product, i.e.,
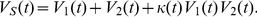
(1)


The proportionality coefficient 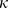 encodes the spatiotemporal information of the input signals, including the input locations and the input arrival times. In addition, we demonstrate that the coefficient 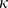 is nearly independent of the input strengths. Because the correction term 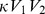 to the linear summation of 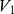 and 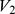 takes a bilinear form, we will refer to the rule (1) as the bilinear spatiotemporal dendritic integration rule. In the remainder of the article, unless otherwise specified, all the membrane potentials will be referred to those measured at the soma.

We note that our bilinear integration rule is consistent with recent experimental observations [3]. In the experiments [3], the rule was examined at the time when the excitatory postsynaptic potential (EPSP) measured at the soma reaches its peak for a pair of excitatory and inhibitory inputs elicited concurrently. We demonstrate that our bilinear integration rule is more general than that in Ref. [3]: (i) our rule holds for a pair of excitatory and inhibitory inputs that can arrive at different times; (ii) our rule is also valid at any time and is not limited to the peak time of the EPSP; (iii) our rule is general for all types of paired synaptic input integration, including excitatory-inhibitory, excitatory-excitatory and inhibitory-inhibitory inputs.

Our bilinear integration rule is derived from the two-compartment passive cable model. We then validate the rule in a biologically realistic pyramidal neuron model with active ion channels embedded. The simulation results from the realistic model are consistent with the rule derived from the passive cable model. We further validate the rule in electrophysiological experiments in rat hippocampal CA1 pyramidal neurons. All of our results suggest that the form of the bilinear integration rule is preserved in the presence of active dendrites.

As mentioned previously, there are thousands of synaptic inputs received by a neuron in the brain. We therefore further apply our analysis to describe the dendritic integration of multiple synaptic inputs. We demonstrate that the spatiotemporal dendritic integration of all synaptic inputs can be decomposed into the sum of all possible pairwise dendritic integration, and each pair obeys the bilinear integration rule (1), i.e.,
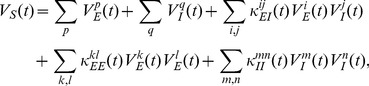
(2)where 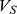 denotes the SSP, 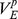 denotes the 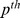 individual EPSP, 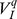 denotes the 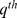 individual inhibitory postsynaptic potential (IPSP), 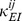, 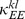, and 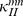 are the corresponding proportionality coefficients with superscripts denoting the index of the synaptic inputs. We then confirm the bilinear integration rule (2) numerically using realistic neuron modeling. The decomposition of multiple inputs integration in rule (2) leads to a graph representation of the dendritic integration. Each node in the graph corresponds to a synaptic input location, and each edge connecting two nodes represents the bilinear term for a pair of synaptic inputs given at the corresponding locations. This graph evolves with time, and is all-to-all connected when stimuli are given at all synaptic sites simultaneously. However, based on simulation results and experimental observations, we can estimate that there are only a small number of activated synaptic integration, or edges in the graph, within a short time interval. Therefore, the graph representing the dendritic integration can indeed be functionally sparse.

Finally, we comment that, in general, it is theoretically challenging to analytically describe the dynamical response of a neuron with dendritic structures under time-dependent synaptic conductance inputs. One simple approach to circumvent this difficulty is to analyze the steady state of neuronal input-output relationships by assuming that both the synaptic conductance and the membrane potential are constant [3], [12]. Such analyses can be applied to study dendritic integration, but they usually oversimplify the description of the spatial integration, and fail to describe the temporal integration. Another approach to circumvent the difficulty is to study the cable model [14], [15] analytically or numerically. For the subthreshold regime, in which voltage-gated channels are weakly activated, the dendrites can be considered as a passive cable. Along the cable, the membrane potential is linearly dependent on injected current input. This linearity enables one to use the Green's function method to analytically obtain the membrane potential with externally injected current. In contrast, the membrane potential depends nonlinearly on the synaptic conductance input [12]. This nonlinearity greatly complicates mathematical analyses. Therefore, in order to solve the cable model analytically, one usually makes the approximation of constant synaptic conductance [16], [17]. The approximation can help investigate some aspects of dendritic integration, however, the approximation in such a case is not sufficiently realistic because the synaptic conductances *in vivo* are generally time-dependent. On the other hand, one can study the dendritic integration in the cable model numerically. The compartmental modeling approach [14] enables one to solve the cable model with time-dependent synaptic inputs. This approach has been used to investigate many aspects of dendritic integration. For instance, it was discovered computationally that dendritic integration of excitatory inputs obeys a certain qualitative rule, i.e., EPSPs are first integrated nonlinearly at individual branches before summed linearly at the soma [18], [19], which was verified later in experiments [20], [21]. Clearly, the computational approach can help gain insights into various phenomena of spatiotemporal dynamics observed at the dendrites, however, a deep, comprehensive understanding often requires analytical approaches. Note that this point has also been emphasized in Ref. [22]. Here, our analytical asymptotic method can solve the cable model with time-dependent synaptic inputs analytically and reveal a precise quantitative spatiotemporal dendritic integration rule, as will be further illustrated below.

## Results

We first study the dendritic integration of a pair of excitatory and inhibitory inputs (E-I), a pair of excitatory inputs (E-E), and a pair of inhibitory inputs (I-I) case by case. In each case, we first analytically derive the bilinear integration rule from the two-compartment passive cable model, and then validate the bilinear integration rule using the realistic model of a pyramidal neuron with both active channels and dendritic branches; we further validate the bilinear integration rule in electrophysiological experiments in rat CA1 pyramidal neurons. We then derive the bilinear integration rule for multiple excitatory and inhibitory inputs, and validate this rule in the simulation. Based on our bilinear integration rule for multi-inputs, we finally propose a graph representation of dendritic integration.

### Bilinear Rule for E-I Integration

We begin to study the spatiotemporal dendritic integration of a pair of excitatory and inhibitory inputs. An analytical derivation of the bilinear integration rule is described in the section of Derivation of the Rule. The details of the cable model used in the derivation can be found in the section of Materials and Methods. The validation of the bilinear integration rule using the realistic neuron modeling and electrophysiological experiments is described in the section of Validation of the Rule. The spatial dependence of the coefficient 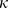 in the rule is described in the section of Spatial Dependence of 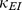.

#### Derivation of the rule

Most neurons possess complicated dendritic morphology, however, for simplicity, we start to investigate the spatiotemporal dendritic integration rule with an idealized two-compartment passive cable model, in which a spherical soma is connected to an unbranched cylindrical dendrite with finite length 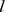 and diameter 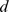. The distance between a dendritic location and the soma is denoted by 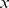. Given an excitatory input at location 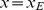 and at time 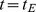, and an inhibitory input at location 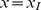 and at time 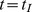, the membrane potential dynamics is governed by
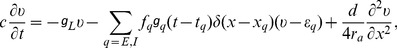
(3)


where 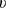 is the membrane potential with respect to the resting potential on the dendrite, 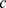 is the membrane capacitance per unit area, 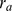 is the axial resistivity, and 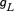 is the leak conductance per unit area. The excitatory and inhibitory input strengths 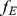 and 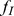 control the amplitude of EPSP and IPSP, respectively. The variables 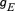 and 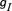 denote the unitary excitatory and inhibitory conductances per unit area with their peak value normalized to unity [described by Equation (29)]. 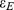 and 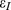 are the corresponding reversal potentials, respectively. The values of the parameters in the model can be found in the section of Materials and Methods.

By assuming that one dendritic end is sealed and the other dendritic end connects to the spherical soma, the boundary conditions are given by
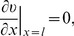
(4)

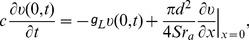
(5)where 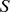 is the surface area of the soma. The initial condition is simply set as
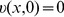
(6)for a neuron at its resting state.

For the physiological regime, the corresponding synaptic input strengths, 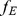 and 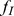, are relatively small in the model [Equations (3)–(6)]. To be specific, for the amplitude of an EPSP less than 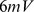 and the amplitude of an IPSP less than 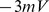, the corresponding strengths 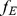 and 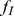 are considered to be small. Therefore, we can represent the membrane potential as an asymptotic series in powers of 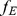 and 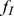 as follows:
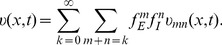
(7)


Substituting Equation (7) into Equations (3)–(6), order by order, we can obtain its asymptotic solutions 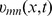. The solutions to the second order (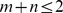) are described below (see the section of Materials and Methods for a detailed calculation). For the zeroth order, we have
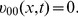
(8)


The solution corresponds to the fact that the membrane potential response remains at its resting state when there is no stimuli presented (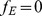, 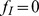). The first order excitation 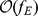 is
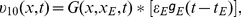
(9)where ‘
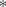
’ denotes convolution in time, and 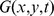 is the Green's function of the cable equation given a 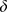 impulse input (the analytical expression is presented in the section of Materials and Methods). Note that the input 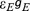 at 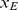 can be viewed as the synaptic current when the local membrane potential is maintained at the resting state. The second order of excitation 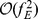 is
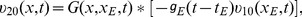
(10)where the expression of 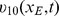 is given in Equation (9). Note that the input 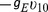 at 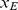 can be viewed as the synaptic current, which is the product of the local conductance 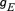 with the first order local membrane potential 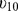. Similarly, we have the first and second order inhibition 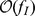 and 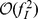:
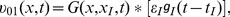
(11)

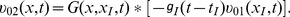
(12)


For the order of 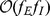, we have
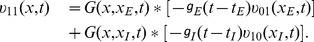
(13)where 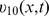 and 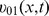 are given by Equations (9) and (11), respectively. On account of the fact that 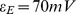 (relative to the resting potential) is nearly an order of magnitude larger than 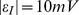, 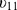 in Equation (13) can be further simplified as
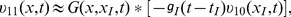
(14)which indicates that the nonlinear integration effect mainly originates from the outward synaptic current, i.e., 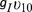, induced by the first order EPSP measured at the inhibitory input site 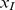, i.e., 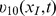.

**Figure 1:**
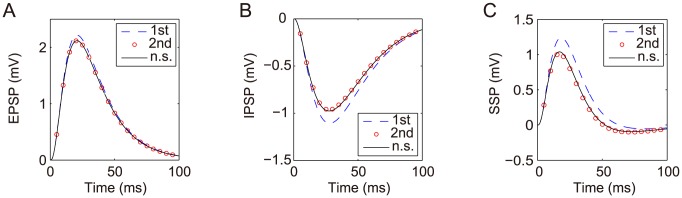
Asymptotic solutions of various orders for the two-compartment passive cable model. Asymptotic solutions for (A) EPSP, (B) IPSP, and (C) SSP in comparison with numerical simulations of Equation (3). The blue dashed line is the first order approximation. The red circle is the second order approximation. The black solid line is the numerical solution of the full Equation (3). The stimuli are given at the location 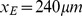 and 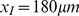. Physiological parameters in the simulation can be found in the section of Materials and Methods.

Numerical simulation of the cable model indicates that the second order asymptotic approximation is sufficiently accurate in capturing the model's solution of physiologically realistic membrane potentials, as demonstrated in Fig. 1. Therefore, if only an individual excitatory input is given [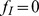 in Equation (3)], the corresponding EPSP measured at the soma, denoted by 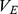, can be approximated by
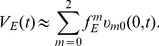
(15)


Similarly, if only an individual inhibitory input is given [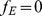 in Equation (3)], the corresponding IPSP measured at the soma, denoted by 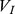, can be approximated by
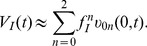
(16)


If both the excitatory and inhibitory inputs are given at 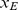 and 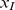, the corresponding SSP measured at the soma, denoted by 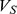, can be approximated by
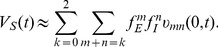
(17)


We define the difference between the SSP, and the linear summation of EPSP and IPSP as
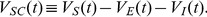
(18)


From Equations (15), (16) and (17), we have
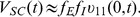
(19)


If 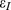 is set to be at the resting potential, we can show that the value of 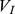 indicating hyperpolarization vanishes, while the value of 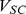 stays nearly the same. Therefore, 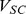 is mainly caused by the shunting effect and thus is referred to as the shunting component (SC). In our analysis, the SC is the leading order of the nonlinear integration between excitation and inhibition. If we further define the shunting coefficient 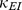 as
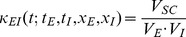
(20a)

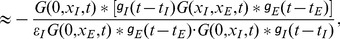
(20b)


then 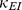 is nearly independent of the amplitude of EPSP and IPSP, because the input strengths 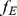 and 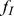, which determine the amplitudes of EPSP and IPSP, cancel each other in both denominator and numerator in Equation (20b).

From Equation (20a), we have the following spatiotemporal dendritic integration rule
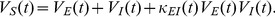
(21)


**Figure 2:**
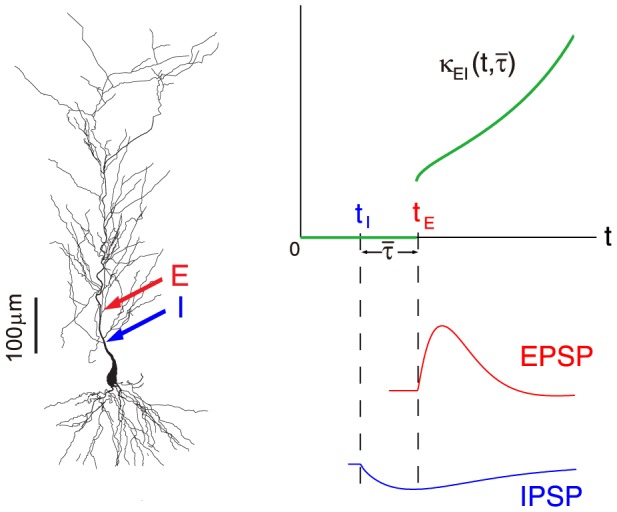
Description of 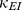 as a function of time 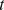 and stimulus arrival difference 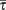 for a fixed pair of excitatory and inhibitory input locations. Left, a morphological plot of the realistic neuron model. The excitatory and inhibitory input locations are indicated by arrows. Right, (lower) an IPSP arrives at the soma earlier than an EPSP. The arrival times are indicated by vertical dashed lines. (upper) The shunting coefficient 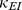 remains at zero until the EPSP arrives at 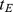.

The shunting coefficient 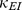 is nearly independent of the amplitude of EPSP and IPSP. In addition, 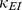 depends on the location of excitatory and inhibitory inputs 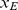 and 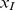. For a fixed pair of excitatory and inhibitory input locations, 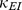 is a function of both time 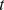 and the arrival time difference between the excitatory and inhibitory input 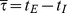, as illustrated in Fig. 2.

#### Validation of the Rule

As the bilinear integration rule (21) is derived from the idealized passive neuron model, we need to investigate its validity for a realistic neuron, which has active ion channels embedded in its tree-like dendrites.

We first perform the simulation of a biologically realistic pyramidal neuron with active channels (morphology shown in Fig. 2). The details of the model and the related computational method can be found in the section of Materials and Methods. The simulation results are summarized below.

**Figure 3:**
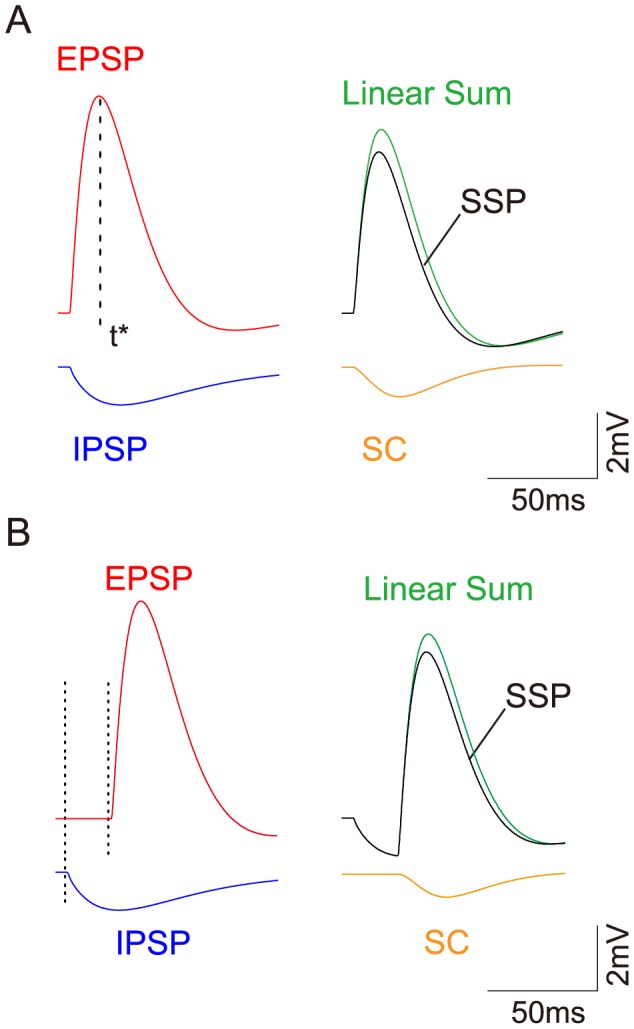
An example of EPSP, IPSP, SSP, SC, and the corresponding linear sum. (A) The EPSP and the IPSP are elicited concurrently. Here 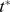 denotes the time when EPSP reaches its peak value. (B) the IPSP is elicited 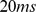 before the EPSP. The results are obtained in the realistic pyramidal neuron model simulation which is described in detail in the section of Materials and Methods. The excitatory input is given at the location 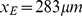 and the inhibitory input is given at the location 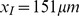.

**Figure 4:**
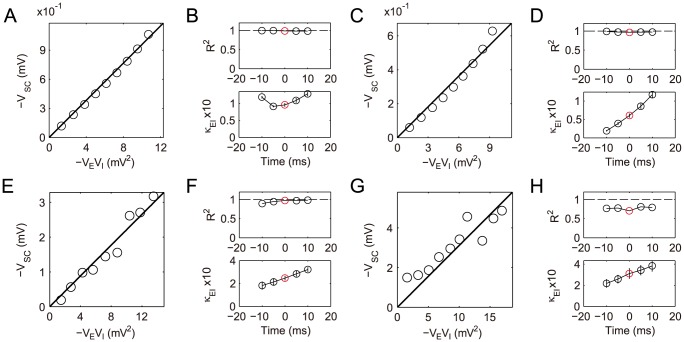
Dendritic integration of a pair of excitatory and inhibitory inputs. (A–D) Simulation results with the excitatory input given at the location 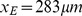 and the inhibitory input given at the location 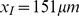. (A) The SC amplitude is plotted against the product of EPSP amplitude and IPSP amplitude, at the time when EPSP reaches its peak, i.e., 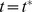 (Note that 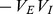 and 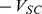 are plotted). Varying 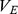 less than 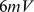 and varying 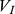 less than 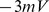, it can be seen that 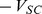 increases linearly with 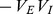. (B) Dendritic integration in the time interval 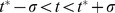, where 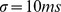. (upper) 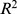 for the goodness of the linear fitting of 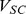 vs. 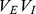 at different times. (lower) The shunting coefficient 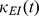 (in the unit of 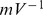) as the slope of the linear fitting is plotted at different times. The error bar indicates 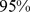 confidence interval (The error bars are relatively small and are within the circles). The circle marked by red indicates the case in (A). (C–D) The same as (A–B) except that the IPSP is elicited 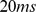 before the EPSP. (E–H) Experimental results with the excitatory input given at the location 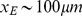 and the inhibitory input given at the location 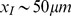. (E–F) for concurrent inputs and (G–H) for nonconcurrent inputs that the IPSP is elicited 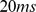 earlier than the EPSP.

For the case of concurrent inputs (Here, we use “concurrent” to denote the case when 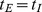), as shown in Fig. 3A, when the excitatory and inhibitory inputs are elicited concurrently at different locations on the dendritic trunk, the SSP is found to be always smaller than the linear sum of the EPSP and the IPSP when elicited separately. In this case, the bilinear integration rule (21) holds at the time 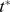 when the EPSP reaches its peak value. We can vary 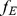 to control the amplitude of EPSP (less than 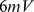) and vary 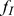 to control the amplitude of IPSP (less than 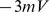). For fixed input strengths 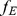 and 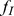, we obtain the set of time courses of the EPSP, the IPSP, and the corresponding SSP. Using 9 sets of such data with different input strengths, we find that the SC amplitude 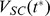 depends linearly on the product of the EPSP and IPSP amplitudes, i.e., 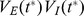. The excellent linear fitting in Fig. 4A shows that the slope 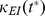 is independent of the amplitude of EPSP and IPSP. This result is consistent with the experimental observation [3].

For the same case of concurrent inputs, we then calculate 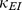 at any time 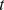, instead of the peak time 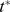. We compute 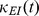 within the interval 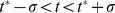, where 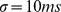. We choose this 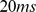 interval because the amplitude of the EPSP is relatively large within that interval. As a consequence, we can avoid a small denominator in calculating 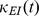 and improve the numerical accuracy. At each fixed time 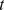, 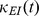 is estimated by linear regression using the same 9 sets of data used to validate the bilinear integration rule at 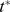. As demonstrated in Fig. 4B, at each time 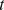, there is a small error bar for the slope 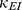 estimation and the 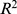 value is very close to 1. Both facts indicate an excellent linear fitting of 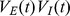 vs. 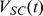. Therefore, the shunting coefficient 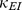 is nearly independent of the amplitude of EPSP (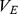) and IPSP (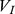) at any time. As expected, the error bar for the slope estimation increases dramatically far away from the peak time due to the fact that the numerical accuracy is low when EPSP and IPSP are small, in particular, when they approach zero. However, the SC amplitude in this case is sufficiently small, and can thus be neglected. Therefore, the bilinear integration rule can naturally be considered valid with 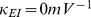.

For the case of nonconcurrent inputs, when the onset of the inhibitory input is 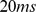 earlier than that of the excitatory input (Fig. 3B), our numerical results show that the bilinear integration rule (21) still holds, as shown in Fig. 4C–D. The rule is also confirmed for any excitatory and inhibitory input locations arbitrarily distributed on the dendritic trees.

We next perform electrophysiological experiments to validate our bilinear integration rule (21). The details of the experimental procedure can be found in the section of Materials and Methods. In experiments, the excitatory input is given at 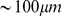 with the EPSP amplitude less than 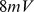 and the inhibitory input is given at 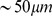 with the IPSP amplitude less than 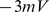. For the case of concurrent inputs, we found that 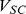 depends linearly on 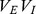 at the time when EPSP reaches its peak, as shown in Fig. 4E, and at a non-peak time, as shown in Fig. 4F. Therefore, the slope 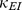 is nearly independent of EPSP and IPSP amplitudes. For the case of nonconcurrent inputs, when the IPSP is elicited 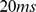 earlier than the EPSP, the linear relationship between 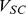 and 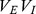 still holds, as shown in Fig. 4G–H, except that the value 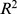 of the regression is smaller (0.77 to 0.81) than those in the concurrent case (0.90 to 0.99). Therefore, it can be seen from the above that, the bilinear integration rule (21) is confirmed in rat hippocampal CA1 pyramidal neurons.

Note that the bilinear integration rule is derived from an idealized passive neuron case. Interestingly, our results from the simulation and experiments demonstrate that the structure of the rule is preserved in the presence of both active channels and dendritic branches.

#### Spatial dependence of 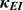


Although we have obtained the form of the bilinear integration rule (21), how the value of the shunting coefficient 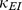 depends on the input location is difficult to analyze directly from its explicit form [Equation (20b)]. Here we investigate the spatial dependence of input location for 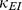, which may partially reveal the way in which 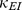 encodes spatial integration information.

**Figure 5:**
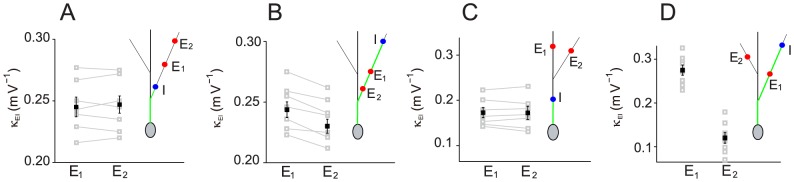
Shunting coefficient 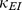 in branched dendrites measured in experiments. The data marked by grey squares were collected from 7 neurons in our experiments, and lines connect data from the same neuron. The data marked by black squares are the average of the data marked by grey squares. The error bar indicates one standard deviation. In all figure panels, the locations of the inhibitory input (I) and excitatory inputs (E1 and E2) are marked by a blue dot and red dots, respectively. The I path is marked by green. (A) The inhibitory input I at an oblique branch: 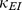 is nearly constant for two distal E1 and E2 on the same branch. (B) As in (A) except that E1 and E2 are more proximal than I. 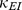 is significantly different at E1 and E2 sites. (C) The inhibitory input I at the trunk: 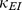 is nearly constant between E1 at the trunk and E2 at the oblique branch. (D) The inhibitory input I at an oblique branch: 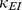 is significantly different between E1 and E2, where E1 is on the same branch as I and E2 is on a different branch.

In our previous study [23], a spatial rule for 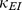 as a function of input locations has been proposed based on a theoretical analysis of a multi-compartment cable model. Under the situation when the excitatory and inhibitory inputs are given concurrently, the spatial profile of 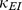 at the time when EPSP reaches its peak is characterized by the following spatial rule: For a fixed inhibitory input location, the I path (marked by green on the dendritic trees in Fig. 5) is defined as the path between the soma and the inhibitory input; Along the I path, 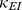 is predicted to increase as the distance between excitatory input location and the soma increases; for any branch (including the trunk) connecting to the I path, 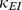 is predicted to be constant for all excitatory input sites on the branch.

The prediction of this spatial 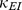 rule is consistent with our electrophysiological experimental results as shown in Fig. 5 (also see Ref. [3]). We next use the spatial 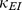 rule to explain these experimental results.

In Fig. 5A and 5B, an inhibitory input is given on an oblique branch and two excitatory inputs are given at two locations on the same oblique branch. The corresponding shunting coefficients 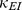 were estimated based on Equation (20a). In our experiment, no significant difference was found between the values of 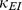 when the two excitatory locations were both distal, as shown in Fig. 5A. This experimental observation can be easily understood by our spatial rule for 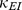 because the two excitatory inputs are on the same branch connected to the I path. By our rule above, the shunting coefficient 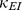 should be the same on such a branch.

In contrast, as shown in Fig. 5B, for two excitatory inputs at proximal locations, 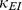 was experimentally found significantly smaller for the excitatory input closer to the soma. This is consistent with our rule because 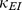 is predicted to be an increasing function of the distance between the excitatory input location and the soma for this case.

In Fig. 5C, an inhibitory input is given on the apical trunk and two distal excitatory inputs are given at either the trunk or a branch. For this case, the 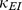 values were found to be nearly constant in our experiment. This is the case in which the two branches where the two excitatory inputs are located connect to the I path with the same branching point. Therefore, this experimental observation can be understood through our rule that all 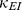 on the two branches are the same as the one at the branching point.

In Fig. 5D, an inhibitory input is given at an oblique branch and two distal excitatory inputs are given at different branches. For this case, the value of 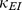 for inhibitory and excitatory inputs located at the same branch was found in our experiment to be significantly larger than the case in which inhibitory and excitatory inputs are located at different branches. For the case when inhibitory and excitatory inputs located at different branches, by our rule, 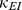 equals to the value at the branching point on the I path. Because this branching point is closer to the soma than the other excitatory input location on the I path, the increase of 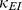 along the I path predicted by our rule explains the experimental observation.

Our theory [23] further predicts that, in principle, it is possible for an inhibitory input located at a branch to shunt the excitatory input on other branches with a large value of 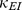. This generalizes the conclusion in Ref. [3], in which the shunting inhibition is mainly confined within the branch where the inhibitory input is located. In Ref. [3], the simulation investigation is focused on the case when inhibitory input is elicited on a branch close to soma. In this case, 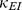 for excitatory inputs on different branches is found to be small in simulation. This observation is consistent with our rule. According to our rule, the value of 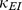 for an excitatory input on the branch is equal to that for excitatory input on the branching point connected to the I path. Due to the branching point being very close to the soma in their simulation, 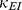 can be small. In general, when the inhibitory input is elicited at a location far from the soma, for the excitatory input on some other distal dendritic branch, the distance between the branching point and soma can be large, which leads to a large value of 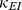.

### Bilinear Rules for E-E & I-I Integration

So far we have addressed the dendritic integration for a pair of excitatory and inhibitory inputs. A natural question arises: how does a neuron integrate a pair of time-dependent synaptic conductance inputs with identical type?

The dendritic integration of excitatory inputs has been extensively investigated in experiments (reviewed in Ref. [1]), yet a precise quantitative characterization is still lacking. According to our idealized cable model, given a pair of excitatory inputs with input strengths 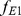 and 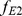 at locations 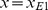 and 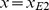 and at times 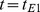 and 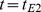, the dynamics of the membrane potential on the dendrite is governed by the following equation:
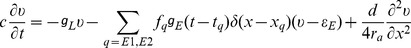
(22)


with the initial and boundary conditions the same as given in Equations (4)–(6). Similarly, we can represent its solution as an asymptotic series and solve it order by order to obtain the following bilinear integration rule:
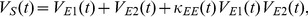
(23)


where 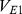 and 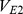 are EPSPs induced by two individual excitatory inputs, and 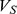 is the SSP when the two excitatory inputs are present. Similar to the case of a pair of excitatory and inhibitory inputs, the shunting coefficient 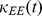 only depends on the excitatory input locations and the input time difference. It does not depend on the EPSPs' amplitudes. Here 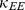 will still be referred to as a shunting coefficient because the origin of the nonlinear integration for the paired excitatory inputs is exactly the same as that for the paired excitatory and inhibitory inputs from the passive cable model.

**Figure 6:**
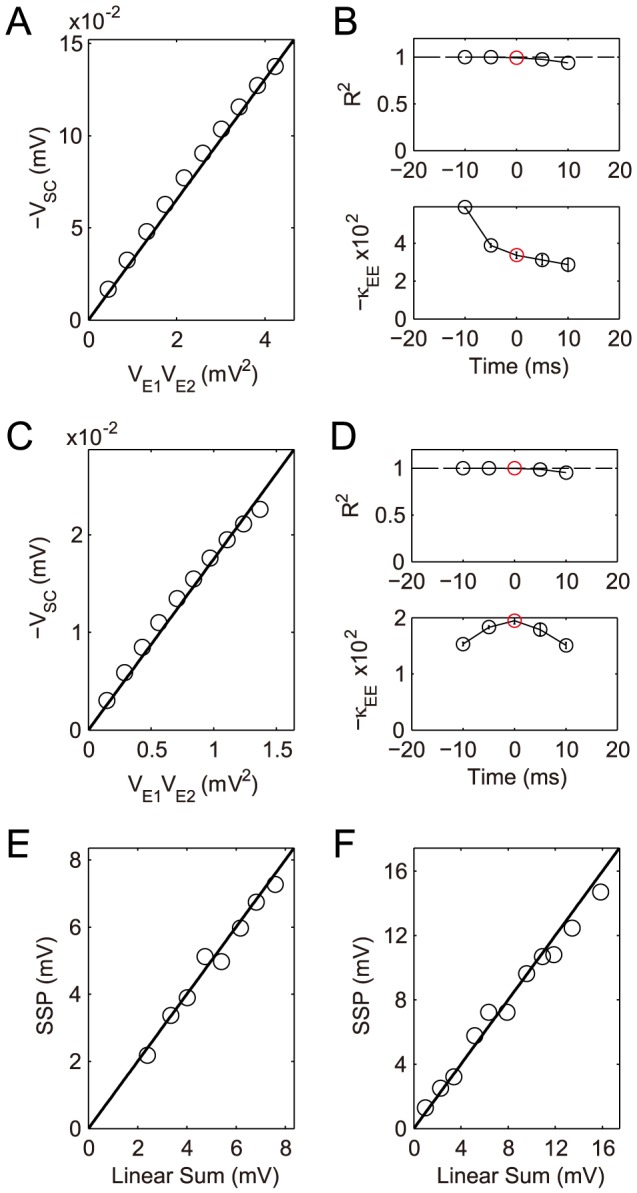
Dendritic integration of a pair of excitatory inputs. (A–D) Simulation results with two excitatory inputs given at the location 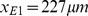 and 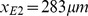. (A) The SC amplitude is plotted against the product of the two EPSP amplitudes, at the time 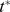 when one of the EPSPs reaches its peak (Note that 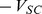 is plotted). Varying 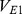 and 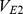 less than 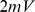, it can be seen that 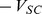 increases linearly with 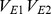. (B) Dendritic integration in the time interval 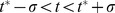, where 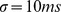. (upper) 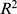 for the goodness of the linear fitting of 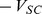 vs. 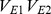 at different times. (lower) The shunting coefficient 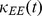 (in the unit of 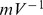) as the slope of the linear fitting is plotted at different times. The error bar indicates 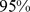 confidence interval (The error bars are relatively small and are within the circles). The circle marked by red indicates the case in (A). (C–D) The same as (A–B) except that one of the EPSPs is elicited 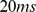 earlier than the other. (E–F) Our experimental result shows the nearly linear summation for (E) a pair of concurrent excitatory inputs and (F) nonconcurrent excitatory inputs with arrival time difference 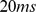, when two excitatory inputs are given at the location 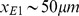 and at 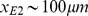.

The bilinear integration rule (23) is found to be consistent with the numerical results obtained using the same realistic pyramidal neuron model as the one used in the section of Bilinear Rule for E–I Integration. For a pair of excitatory inputs with their locations fixed on the dendritic trunk, the rule holds when the amplitude of each EPSP is less than 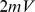. For the case of concurrent inputs, at the time 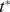 when one of the EPSPs reaches its peak value
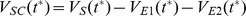
is found to be linearly dependent of 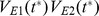, as shown in Fig. 6A. This linear relationship indicates 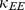 is independent of the amplitudes of the two EPSPs. In addition, as shown in Fig. 6B, the bilinear integration rule is numerically verified in the time interval 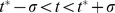, for 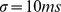, within which the amplitude of EPSPs are relatively large. For the case of nonconcurrent inputs, the bilinear integration rule is also numerically verified in the same way, as shown in Fig. 6C–D.

**Figure 7:**
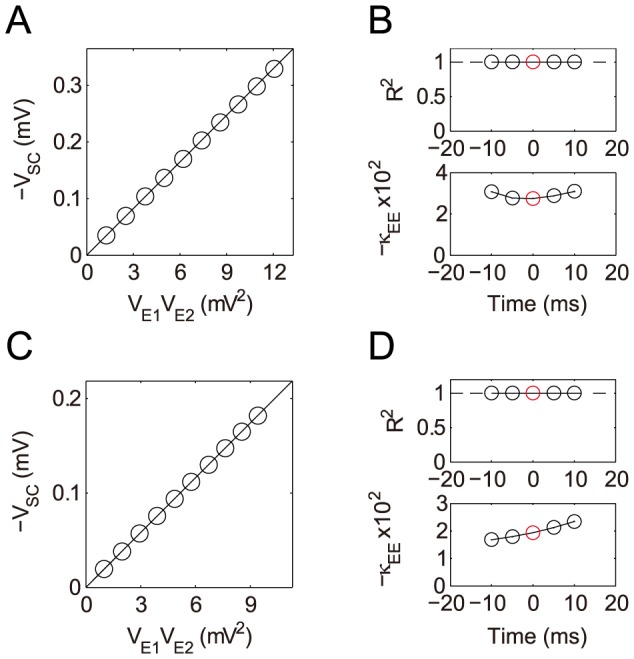
Integration of a pair of excitatory inputs on passive dendrites. Simulation results with two excitatory inputs given at the location 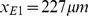 and 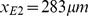. (A) The SC amplitude is plotted against the product of the two EPSP amplitudes, at the time 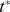 when one of the EPSPs reaches its peak (Note that 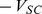 is plotted). (B) Dendritic integration in the time interval 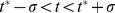, where 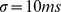. (upper) 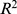 for the goodness of the linear fitting of 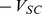 vs. 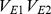 at different times. (lower) The shunting coefficient 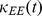 (in the unit of 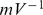) as the slope of the linear fitting is plotted at different times. The error bar indicates 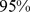 confidence interval (The error bars are relatively small and are within the circles). The circle marked by red indicates the case in (A). (C–D) The same as (A–B) except that one of the EPSPs is elicited 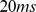 earlier than the other.

In addition, we find that when the input strengths become sufficiently strong so as to make the depolarized membrane potential too large, i.e. 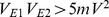, there is a deviation from the bilinear integration rule (23). This deviation can be ascribed to the voltage-gated ionic channel activities in our realistic pyramidal neuron model. After blocking the active channels, the rule becomes valid with a different value of 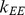 for large EPSPs amplitudes, as shown in Fig. 7. However, we note that, regardless of input strengths, the amplitude of SC is always two orders of magnitude smaller than the amplitude of SSP. Therefore, the integration of two excitatory inputs can be naturally approximated by the linear summation of two individual EPSPs, i.e. 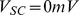. We then perform electrophysiological experiments with a pair of excitatory synaptic inputs to confirm the linear summation. As expected, this linear summation is also observed in our experiments for both concurrent and nonconcurrent input cases, as shown in Fig. 6E and 6F, respectively. Note that, the linear summation is also consistent with experimental observations as reported in Ref. [24].

**Figure 8:**
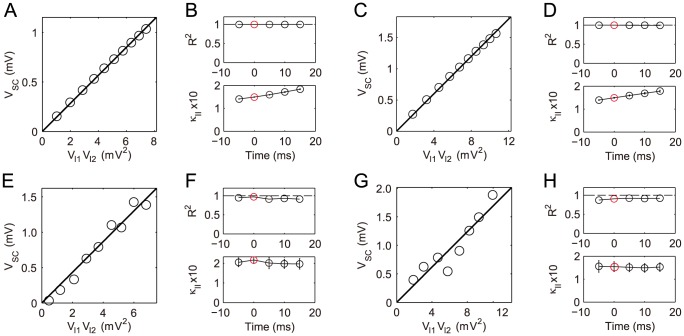
Dendritic integration of a pair of inhibitory inputs. (A–D) Simulation results. Two inhibitory inputs are given at the location 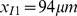 and at 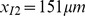. (A) The SC amplitude is plotted against the product of the two IPSP amplitudes, at the time 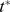 when one of the IPSPs reaches its peak. Varying 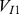 and 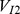 less than 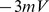, it can be seen that 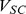 increases linearly with 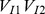. (B) Dendritic integration in the time interval 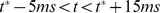. (upper) 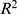 for the goodness of the linear fitting of 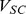 vs. 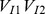 at different times. (lower) The shunting coefficient 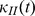 (in the unit of 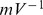) as the slope of the linear fitting is plotted at different times. The error bar indicates 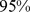 confidence interval (The error bars are relatively small). The circle marked by red indicates the case in (A). (C–D) The same as (A–B) except that one of the IPSPs is elicited 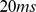 earlier than the other. (E–H) Experimental results with two inhibitory inputs given at the location 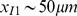 and at 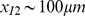. (E–F) for concurrent inhibitory inputs and (G–H) for nonconcurrent inhibitory inputs with arrival time difference 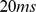.

Similarly, for a pair of inhibitory inputs, we can arrive at the following bilinear integration rule from the cable model:
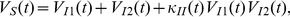
(24)where 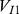 and 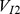 are IPSPs induced by two individual inhibitory inputs, and 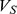 is the SSP when the two inhibitory inputs are present. Here, 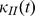 is the shunting coefficient that is independent of the IPSPs amplitudes but is dependent on the input time difference and input locations. The above bilinear integration rule (24) is consistent with our numerical results using the realistic pyramidal neuron model, as shown in Fig. 8A–D. Our electrophysiological experimental observations further confirm this rule, as shown in Fig. 8E–H.

### Bilinear Rule for Multi-input Integration

In the previous sections, we have discussed the integration of a pair of synaptic inputs. *In vivo*, a neuron receives thousands of excitatory and inhibitory inputs from dendrites [2]. Therefore, we now address the question of whether the integration rule derived for a pair of synaptic inputs can be generalized to the case of multiple inputs.

Our theoretical analysis shows that, for multiple inputs, the SSP can be approximated by the linear sum of all individual EPSPs and IPSPs, plus the bilinear interactions between all the paired inputs with shunting coefficients 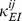, 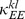, and 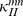 respectively (the superscript labels the synaptic inputs), i.e.,
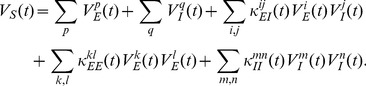
(25)


**Figure 9:**
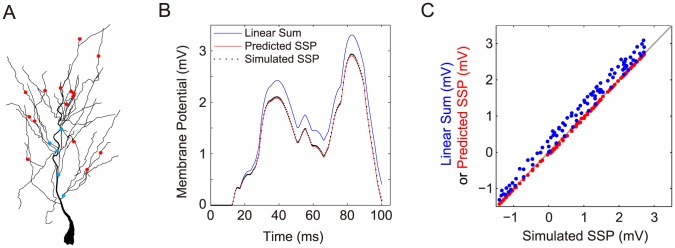
Dendritic integration of multiple synaptic inputs. (A) Distribution of 15 excitatory inputs (red dots) and 5 inhibitory inputs (blue dots) at the dendritic arbor of the realistic pyramidal neuron model. (B) One trial of membrane potential obtained by setting the arrival time of each stimulus randomly distributed from 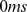 to 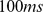. The SSP (black dots) from the simulation of the realistic neuron model nearly overlaps with the SSP (red) predicted by the bilinear integration rule (25) while deviating from the trace of the direct linear summation of all postsynaptic potentials elicited separately (blue). (C) The direct linear sum (blue) and the SSP (red) predicted by rule (25) are plotted against the SSP from the simulation of the realistic neuron model. Here, the data are points on the corresponding curves from ten trials sampled uniformly from 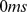 to 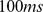. For comparison, the slope of the grey line is unity. It can be observed that the red dots fall on the grey line. This indicates that the predicted SSP is equal to the simulated SSP at any time.

We next validate the rule (25) using the realistic pyramidal neuron model. It has been reported that, for a CA1 neuron, inhibitory inputs are locally concentrated on the proximal dendrites while excitatory inputs are broadly distributed on the entire dendrites [25]. Based on this observation, we randomly choose 15 excitatory input locations and 5 inhibitory input locations on the model neuron's dendrites (Fig. 9A). In the simulation, all inputs are elicited starting randomly from 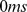 to 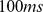. In order to compare Equation (25) with the SSP simulated in the realistic neuron model, we first measure 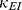, 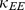, and 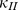 pair by pair for all possible pairs. We then record all membrane potential traces 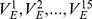 and 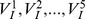 induced by the corresponding individual synaptic inputs. Our results show that the SSP measured from our simulation is indeed given by the bilinear integration rule (25), as shown in Fig. 9B and 9C. In contrast, the SSP in our numerical simulation deviates significantly from the linear summation of all individual EPSPs and IPSPs.

### Graph Representation of Dendritic Integration

**Figure 10:**
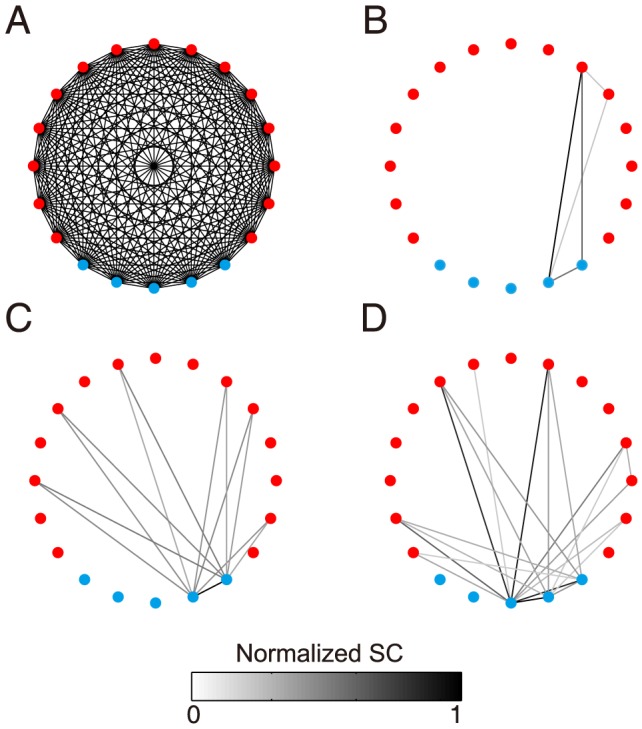
Graph representation of dendritic integration. (A) A complete dendritic graph with 15 excitatory inputs (red) and 5 inhibitory inputs (blue). (B–D) Activated dendritic graph at time 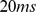, 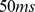 and 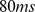, respectively. The color of an edge in (B–D) denotes the normalized SC value. Data are collected from simulations in Fig. 9.

According to our bilinear integration rule (25), the dendritic integration of multiple synaptic inputs can be decomposed into the summation of all possible pairwise dendritic integration. Therefore, we can map dendritic computation in a dendritic tree onto a graph. Each dendritic site corresponds to a node in the graph and the corresponding shunting component is mapped to the weight of the edge connecting the two nodes. We refer to such a graph as a dendritic graph. The dendritic graph is an all-to-all connected graph if all stimuli are given concurrently (Fig. 10A). However, the dendritic integration for all possible pairs of synaptic inputs is usually not activated concurrently in realistic situations. For instance, if the arrival time difference between two inputs is sufficiently large, there is no interaction between them. The activated level of the nonlinear dendritic integration for a pair of synaptic inputs can be quantified by the SC amplitude—the weight of the edge in the graph. The simulation result shows that the number of activated edges at any time is relatively small on the dendritic graph (Fig. 10B–D), compared with the total number of edges on the all-to-all connected graph (Fig. 10A). Therefore, for the case of a hippocampal pyramidal neuron, the dendritic graph could be functionally sparse in time. The functional sparsity of a dendritic graph may also exist in neocortical pyramidal neurons. *In vivo*, a cortical pyramidal neuron receives about 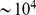 synaptic inputs [26]. Most of them are from other cortical neurons [27], [28], which typically fire about 10 spikes per second in awake animals [29], [30]. Thus, the neuron can be expected to receive 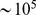 synaptic inputs per second. The average number of synaptic inputs within 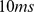 (membrane potential time constants *in vivo*) is 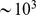. The number of activated dendritic integration pairs within the 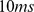 interval is 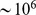, which is relatively small compared with the total possible synaptic integration pairs 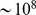. Therefore, the activated integrations or edges in the dendritic graph within a short time window can be indeed functionally sparse (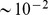). In general, the neuronal firing rates vary across different cell types, cortical regions, brain states and so on. Therefore, based on the above estimate, in an average sense, the graph of dendritic integration is functionally sparse.

## Discussion

Our bilinear dendritic integration rule (21) is consistent with the rule previously reported [3], but is more general in the following aspects: (i) Our dendritic integration rule holds at any time and is not limited to the time when the EPSP reaches its peak value. (ii) The rule holds when the two inputs are even nonconcurrent. This situation often occurs because the excitatory and inhibitory inputs may not always arrive at precisely the same time. (iii) The form of the rule can be extended to describe the integration between a pair of excitatory inputs, a pair of inhibitory inputs, and even multiple inputs of mixed-types. The spatiotemporal information of synaptic inputs interaction is coded in the shunting coefficient, which is a function of the input locations and input arrival time difference.

Our bilinear integration rule holds in the subthreshold regime for a large range of membrane potential. When we derive the bilinear rule from the passive cable model, we assume that the input strengths or the amplitudes of membrane potentials require to be small. This assumption forms the basis of the asymptotic analysis, because the second order asymptotic solutions of EPSP, IPSP and SSP converge to their exact solutions as the asymptotic parameters 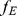 and 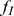 (denoting the excitatory and inhibitory input strengths) approach zero. In general, in the passive cable model, the bilinear rule will be more accurate for small amplitudes of EPSPs and IPSPs than large amplitudes. Importantly, the assumption holds naturally that in the physiological regime when EPSP amplitude is less than 6mV and IPSP amplitude is less than -3mV, 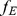 and 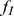 are small 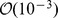. However, even for EPSP amplitude close to the threshold, i.e., 10mV, which is unusually large physiologically, we can show that the second order asymptotic solution can still well approximate the EPSP with a relative error less than 5%. Thus the bilinear rule is still valid for large depolarizations near the threshold. The validity of the bilinear rule for large membrane potentials is also confirmed in both simulations and experiments. In particular, in the analysis of our experimental data, to validate the bilinear rule, we have already included all the data when the EPSP amplitude is below and close to the threshold because we have only excluded those data corresponding to the case when a neuron fires.

Our bilinear dendritic integration rule (21) is derived from the passive cable model. However, the simulation results and the experimental observations demonstrate that the form of dendritic integration is preserved for active dendrites. Additional simulation results show that for the same input locations, the shunting coefficients are generally larger on the active dendrites than those on the passive dendrites with all active channels blocked. We also note that the value of 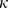 in simulation is different from the value measured in experiments. This difference may arise from the fact that some parameters of the passive membrane properties, such as the membrane leak conductance, may not be exactly the same as those in the biological neuron, and we have only used a limited set of ion channels in simulation compared with those in the biological neuron. In addition, the input locations in the simulation and the experiments are different, which may also contribute to this derivation. However, the bilinear form is a universal feature in both simulation and experiment.

By fixing excitatory input location while varying inhibitory input location, our model exhibits that there exists a region in the distal dendritic trunk within which the shunting inhibition can be more powerful, i.e, a larger 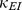, than in proximal dendrites. This result is consistent with what is reported in Ref. [31]. Compared with Ref. [31], our work provides a different perspective of dendritic computation. In their work, the multiple inhibitory inputs can induce a global shunting effect on the dendrites. However, if we focus on the shunting effect only at the soma instead of the dendrites, our theory shows that all the interactions among multiple inputs can then be decomposed into pairwise interactions, as described by the bilinear integration rule (25). In addition, in this work, we focus on the somatic membrane potential that is directly related to the generation of an action potential. However, it is also important to investigate the local integration of membrane potentials measured at a dendritic site instead of that measured at the soma. Asymptotic analysis of the cable model can show that our bilinear integration rule is still valid for the description of the integration on the dendrites. On the dendrites, the broadly distributed dendritic spines with high neck resistances [32], [33] will filter a postsynaptic potential to a few millivolts on a branch [34], [35]. Within this regime our bilinear integration rule is valid. Note that our rule may fail to capture the supralinear integration of synaptic inputs measured on the dendrites during the generation of a dendritic spike [36]. However, if the integration is measured at the soma, our rule remains valid even when there is a dendritic spike induced by a strong excitatory input and an inhibitory synaptic input on different branches [3].

The bilinear integration rule (25) can help improve the computational efficiency in a simulation of neuronal network with dendritic structures. By our results, once the shunting coefficients for all pairs of input locations are measured, we can predict the neuronal response at the soma by the bilinear integration rule (25). By taking advantage of this, one can establish library-based algorithms to simulate the membrane potential dynamics of a biologically realistic neuron. An example of a library-based algorithm can be found in Ref. [37]. To be specific, based on the full simulation of a realistic neuron model, we can measure the time-dependent shunting coefficient as a function of the arrival time difference and input locations for all possible pairs of synaptic inputs and record them in a library in advance. For a particular simulation task, given the specific synaptic inputs on the dendrites, we can then search the library for the corresponding shunting coefficients to compute the neuronal response according to the bilinear integration rule (25) directly. In such a computational framework, one can avoid directly solving partial differential equations that govern the spatiotemporal dynamics of dendrites and greatly reduces the computational cost for large-scale simulations of networks of neurons incorporating dendritic integration.

## Materials and Methods

### Ethics Statement

The animal-use protocol was approved by the Animal Management Committee of the State Key Laboratory of Cognitive Neuroscience & Learning, Beijing Normal University (Reference NO.: IACUC-NKLCNL2013-10).

### The Cable Model

We consider an idealized passive neuron whose isotropic spherical soma is attached to an unbranched cylindric dendrite with finite length 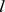 and diameter 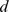. Each small segment in the neuron can be viewed as an RC circuit with a constant capacitance and leak conductance density [11], [38]. The current conservation within a segment 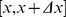 on the dendrite leads to
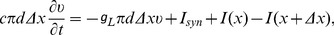
(26)where 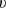 is the membrane potential with respect to the resting potential on the dendrite, 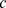 is the membrane capacitance per unit area, and 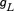 is the leak conductance per unit area. Here, 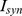 is the synaptic current given by:
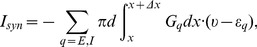
(27)where 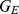 and 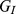 are excitatory and inhibitory synaptic conductance per unit area and 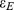 and 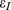 are their reversal potentials, respectively. When excitatory inputs are elicited at 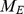 dendritic sites and inhibitory inputs are elicited at 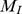 dendritic sites, we have
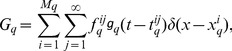
(28)where 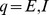. For a synaptic input of type 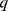, 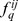 is the input strength of the 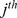 input at the 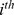 location, 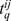 is the arrival time of the 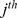 input at the 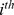 location, 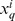 is the 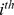 input location. The unitary conductance is often modeled as
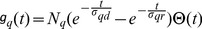
(29)with the peak value normalized to unity by the normalization factor 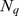, and with 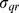 and 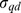 as rise and decay time constants, respectively [38]. Here 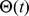 is a Heaviside function. The axial current 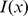 can be derived based on the Ohm's law,
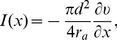
(30)where 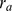 is the axial resistivity. Taking the limit 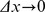, Equation (26) becomes our unbranched dendritic cable model,
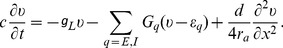
(31)


In particular, for a pair of excitatory and inhibitory inputs with strength 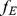 and 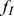 received at 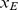 and 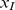, and at time 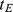 and 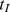, respectively, we have
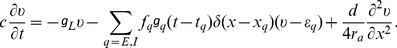
(32)


Similarly, for a pair of excitatory or inhibitory inputs with strengths 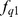 and 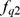 received at 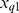 and 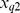, and at time 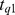 and 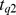 (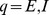), respectively, we have
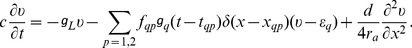
(33)


For the boundary condition of the cable model [Equation (31)], we assume one end of the dendrite is sealed:
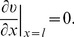
(34)


For the other end connecting to the soma, which can also be modeled as an RC circuit, by the law of current conservation, we have
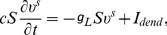
(35)where 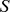 is the somatic membrane area, and 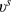 is the somatic membrane potential. The dendritic current flowing to the soma, 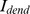, takes the form of Equation (30) at 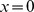. Because the membrane potential is continuous at the connection point
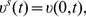
(36)we arrive at the other boundary condition at 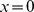:
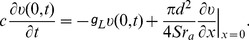
(37)


For a resting neuron, the initial condition is simply set as
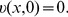
(38)


### Green's Function

In the absence of synaptic inputs, Equation (31) is a linear system. Using a 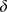 impulse input, its Green's function 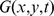 can be obtained from
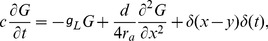
(39)with the following boundary conditions and initial condition,



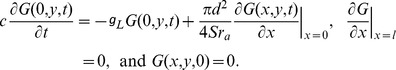



For simplicity, letting 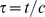, 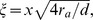 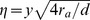, 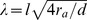, the solution of Equation (39) can be obtained from the following system,
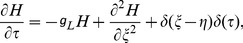
(40)


with rescaled boundary and initial conditions,
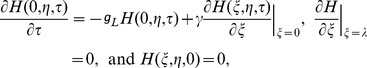



where 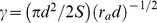. Taking the Laplace transform of Equation (40), we obtain
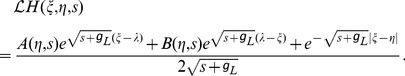
(41)


Combining the two boundary conditions (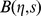 is thus eliminated), we have
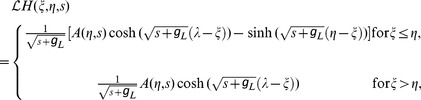
(42)where
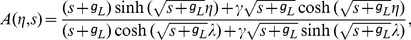
(43)whose denominator is denoted as 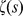 for later discussions. For the inverse Laplace transform, we need to deal with singular points that are given by the roots of 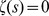. It can be easily verified that these singularities are simple poles and 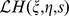 is analytic at infinity. Then 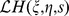 can be written as
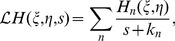
(44)where 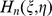 is a constant coefficient in the complex 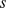 domain, and 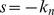 are the singular points. Then taking the inverse Laplace transform of Equation (44), we obtain
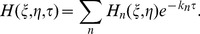
(45)


Now we only need to solve 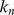 and 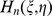 in Equation (45) to obtain the Green's function of Equation (40). We solve the singular points 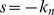 first. Defining 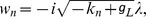 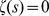 yields
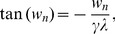
(46)whose roots can be determined numerically. There are solutions for 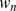 with 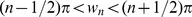 for 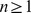 and 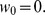 Next, to determine the factors 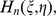 we use the residue theorem for integrals. For a contour 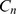 that winds in the counter-clockwise direction around the pole 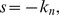 and that does not include any other singular points, the integral of 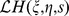 on this contour is given by
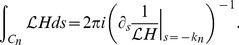
(47)


Using Equations (42–44) and (47), we obtain
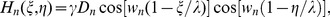
(48)where
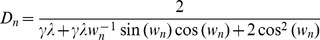
(49)for 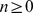. The solution of the original Green's function for Equation (39) can now be expressed as
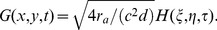
(50)


### Asymptotic Analysis

We first consider the case when a pair of excitatory and inhibitory inputs are received by a neuron. Similar results can be obtained for a pair of excitatory inputs and a pair of inhibitory inputs. For the physiological regime (the amplitude of an EPSP being less than 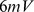 and the amplitude of an IPSP being less than 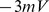), the corresponding required input strengths 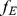 and 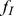 are relatively small. Therefore, given an excitatory input at location 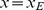 and time 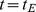, and an inhibitory input at location 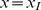 and time 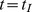, we represent 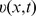 as an asymptotic series in the powers of 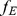 and 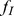,
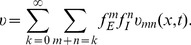
(51)


Substituting Equation (51) into the cable equation (31), order by order, we obtain a set of differential equations. For the zeroth-order, we have
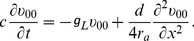
(52)


Using the boundary and initial conditions [Equations (34), (37), and (38)], the solution is simply


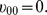
 (53)

For the first order of excitation 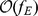, we have
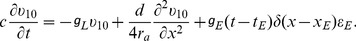
(54)


With the help of Green's function, the solution can be expressed as


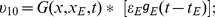
 (55)

here ‘
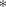
’ denotes convolution in time. For the second order of excitation 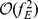, we have
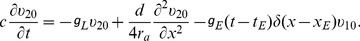
(56)


Because 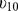 is given by Equation (55), the solution of Equation (56) is
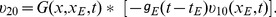
(57)


Similarly, we can have the first and second order inhibitory solutions,
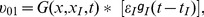
(58)

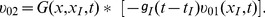
(59)


For the order of 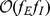, we have
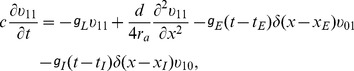
(60)whose solution is obtained as follows,
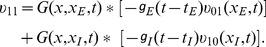
(61)


### Numerical Simulation

For the numerical simulation of the two-compartment passive cable model [Equation (3)], the Crank-Nicolson method [39] was used with time step 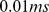 and space step 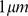. Parameters in our simulation are within the physiological regime [3], [12] with 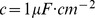, 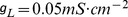, 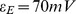, 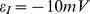, 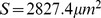, 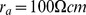, 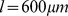, 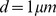. 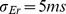, 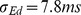, 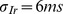, and 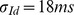. The time constants here were chosen to be consistent with the conductance inputs in the experiment [3].

The realistic pyramidal model is the same as that in Ref. [3]. The morphology of the reconstructed pyramidal neuron includes 200 compartments and was obtained from the Duke-Southampton Archive of neuronal morphology [40]. The passive cable properties and the density and distribution of active conductances in the model neruon were based on published experimental data obtained from hippocampal and cortical pyramidal neurons [18], [19], [34], [41]–[50]. We used the NEURON software Version 7.3 [51] to simulate the model with time step 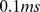.

### Hippocampal Slice Preparation and Electrophysiology

The experimental measurements of summation of EPSPs or IPSPs in single hippocampal CA1 pyramidal cells in the acute brain slice followed a method described in Ref. [3], with some modifications. A brief description of modified experimental procedure is as follows. Acute hippocampal slices (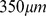 thick) were prepared from Sprague Dawley rats (postnatal day 14–16), using a vibratome (VT1200, Leica). The slices were incubated at 34°*C* for 30 min before transferring to a recording chamber perfused with the aCSF solution (2ml/min; 30–32°*C*). The aCSF contained (in mM) 125 NaCl, 3 KCl, 2 CaCl_2_, 2 MgSO_4_, 1.25 NaH_2_PO_4_, 1.3 sodium ascorbate, 0.6 sodium pyruvate, 26 NaHCO_3_, and 11 D-glucose, and was saturated with gas containing 95% O_2_ and 5% CO_2_ (pH 7.4). Whole-cell recording was made from the soma of CA1 pyramidal cells using glass micropipettes under an upright microscope (BX51WI, Olympus) equipped with the DIC optics and an infrared camera (IR-1000E, DAGE-MTI). The intra-micropipette solution contained (in mM) 145 K-gluconate, 5 KCl, 10 HEPES, 10 disodium phosphocreatine, 4 Mg_2_ATP, 0.3 Na_2_GTP, and 0.2 EGTA (pH 7.3), together with fluorescent dye Alexa Fluor 488 (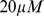, Invitrogen) to visualize the dendritic trees. Pipette resistance was about 3–4 MΩ, and the access resistance during the whole-cell recording was normally less than 20 MΩ. The same method for micro-iontophoretic application of extracelluar glutamate or GABA at the apical dendrite of CA1 pyramidal cells was used to elicit rapid membrane depolarizations (EPSPs) and hyperpolarizations (IPSPs). For all three experimental configurations (EPSP-IPSP, EPSP-EPSP and IPSP-IPSP summation), two micro-iontophoretic pipettes were placed at dendritic locations 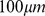 and 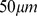 from the soma, respectively, in particular for the EPSP-IPSP summation GABA iontophoretic pipette was always placed at the more proximal location than glutamate iontophoretic pipette was placed. For each recorded cell, an electrode was placed at the soma to set the resting membrane potential to about 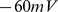 in order to obtain a driving force of 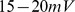 for inhibitory GABA inputs. Electrical signals of individual and summed iontophoretic responses were amplified and filtered at 3 kHz (low pass) by a patch clamp amplifier (MultiClamp 700B, Molecular Devices), digitalized (100 kHz) by an AD-DA converter (Digidata 1440A, Molecular Devices), and acquired by a pClamp 10.3 (Molecular Devices) into a computer for further analysis.

### Data Processing

In order to study the dendritic integration of a pair of excitatory and inhibitory inputs, for fixed input locations and strengths, a moving average technique with time lag 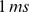 was first applied to smooth each individual trace of the EPSP, IPSP, and SSP recorded in our experiments. After smoothing, we measured the amplitudes of EPSP, IPSP, and SSP at different times, including those when EPSP reached its peak value, and denoted them by 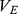, 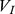, and 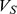, respectively. By varying the excitatory and inhibitory input strengths, we measured values of 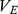, 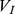 and 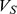. We then constructed a scatter plot of 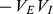 vs. 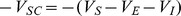 at different times. We divided the range of 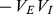 into approximately 10 bins and averaged all the data points 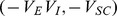 within each bin. The number of bins was chosen to ensure at least 8 data points were used for averaging. However, the qualitative results were not sensitive to the number of bins (e.g, from 6 bins to 16 bins). Using the Curve Fitting Toolbox in Matlab Version 7.14, we finally fitted the averaged data points by a linear function 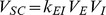, from which the slope 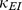 was estimated together with its 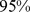 confidence interval. For the dendritic integration of a pair of identical type, the same data processing procedure was followed.

## References

[1] Magee JC (2000) Dendritic integration of excitatory synaptic input. Nature Reviews Neuroscience 1: 181–190. 11257906 10.1038/35044552

[2] Stuart G, Spruston N, Häusser M (2007) Dendrites. Oxford: Oxford University Press.

[3] Hao J, Wang X, Dan Y, Poo M, Zhang X (2009) An arithmetic rule for spatial summation of excitatory and inhibitory inputs in pyramidal neurons. Proc Natl Acad Sci USA 106: 21906–21911. 19955407 10.1073/pnas.0912022106 PMC2799885

[4] Cuntz H, Remme MW, Torben-Nielsen B (2014) The Computing Dendrite. Springer.

[5] Gabbiani F, Krapp HG, Koch C, Laurent G (2002) Multiplicative computation in a visual neuron sensitive to looming. Nature 420: 320–324. 12447440 10.1038/nature01190

[6] David F, Linster C, Cleland TA (2008) Lateral dendritic shunt inhibition can regularize mitral cell spike patterning. Journal of computational neuroscience 25: 25–38. 18060489 10.1007/s10827-007-0063-5

[7] Chacron MJ (2006) Nonlinear information processing in a model sensory system. Journal of Neurophysiology 95: 2933–2946. 16495358 10.1152/jn.01296.2005 PMC5053817

[8] Atallah B, Scanziani M (2009) Instantaneous modulation of gamma oscillation frequency by balancing excitation with inhibition. Neuron 62: 566–577. 19477157 10.1016/j.neuron.2009.04.027 PMC2702525

[9] Vida I, Bartos M, Jonas P (2006) Shunting inhibition improves robustness of gamma oscillations in hippocampal interneuron networks by homogenizing firing rates. Neuron 49: 107–117. 16387643 10.1016/j.neuron.2005.11.036

[10] London M, Häusser M (2005) Dendritic computation. Annu Rev Neurosci 28: 503–532. 16033324 10.1146/annurev.neuro.28.061604.135703

[11] Tuckwell HC (1988) Introduction to Theoretical Neurobiology: Volume 1, Linear Cable Theory and Dendritic Structure, volume 1. Cambridge University Press.

[12] Koch C (2004) Biophysics of computation: information processing in single neurons. Oxford university press.

[13] Zhou D, Li S, Zhang Xh, Cai D (2013) Phenomenological incorporation of nonlinear dendritic integration using integrate-and-fire neuronal frameworks. PloS ONE 8: e53508. 23308241 10.1371/journal.pone.0053508 PMC3538611

[14] Rall W (1964) Theoretical significance of dendritic trees for neuronal input-output relations. In: Reiss R, editor, Neural Theory and Modeling, Standford: Stanford University Press. pp. 73–97.

[15] Rall W (1995) The theoretical foundation of dendritic function: selected papers of Wilfrid Rall with commentaries. MIT press.

[16] Holmes W (1986) A continuous cable method for determining the transient potential in passive dendritic trees of known geometry. Biological cybernetics 55: 115–124. 3801532 10.1007/BF00341927

[17] Timofeeva Y, Cox SJ, Coombes S, Josić K (2008) Democratization in a passive dendritic tree: an analytical investigation. Journal of computational neuroscience 25: 228–244. 18253822 10.1007/s10827-008-0075-9

[18] Poirazi P, Brannon T, Mel BW (2003) Arithmetic of subthreshold synaptic summation in a model ca1 pyramidal cell. Neuron 37: 977–987. 12670426 10.1016/s0896-6273(03)00148-x

[19] Poirazi P, Brannon T, Mel BW (2003) Pyramidal neuron as two-layer neural network. Neuron 37: 989–999. 12670427 10.1016/s0896-6273(03)00149-1

[20] Polsky A, Mel BW, Schiller J (2004) Computational subunits in thin dendrites of pyramidal cells. Nature neuroscience 7: 621–627. 15156147 10.1038/nn1253

[21] Losonczy A, Magee JC (2006) Integrative properties of radial oblique dendrites in hippocampal ca1 pyramidal neurons. Neuron 50: 291–307. 16630839 10.1016/j.neuron.2006.03.016

[22] Segev I, London M (2000) Untangling dendrites with quantitative models. Science 290: 744–750. 11052930 10.1126/science.290.5492.744

[23] Li S, Zhou D, Cai D (2015) Analysis of the dendritic integration of excitatory and inhibitory inputs using cable models. Communications in Mathematical Sciences. In press.

[24] Cash S, Yuste R (1998) Input summation by cultured pyramidal neurons is linear and position-independent. The Journal of neuroscience 18: 10–15. 9412481 10.1523/JNEUROSCI.18-01-00010.1998 PMC6793421

[25] Megas M, Emri Z, Freund T, Gulyas A (2001) Total number and distribution of inhibitory and excitatory synapses on hippocampal ca1 pyramidal cells. Neuroscience 102: 527–540. 11226691 10.1016/s0306-4522(00)00496-6

[26] DeFelipe J, Fariñas I (1992) The pyramidal neuron of the cerebral cortex: morphological and chemical characteristics of the synaptic inputs. Progress in neurobiology 39: 563–607. 1410442 10.1016/0301-0082(92)90015-7

[27] Gruner JE, Hirsch JC, Sotelo C (1974) Ultrastructural features of the isolated suprasylvian gyrus in the cat. Journal of Comparative Neurology 154: 1–27. 4815182 10.1002/cne.901540102

[28] Szentagothai J (1964) The use of degeneration methods in the investigation of short neuronal connexions. Progress in brain research 14: 1–32. 14317758

[29] Steriade M, Deschênes M, Oakson G (1974) Inhibitory processes and interneuronal apparatus in motor cortex during sleep and waking. i. background firing and responsiveness of pyramidal tract neurons and interneurons. Journal of neurophysiology 37: 1065–1092. 4370112 10.1152/jn.1974.37.5.1065

[30] Steriade M (1978) Cortical long-axoned cells and putative interneurons during the sleep-waking cycle. Behavioral and Brain Sciences 1: 465–485.

[31] Gidon A, Segev I (2012) Principles governing the operation of synaptic inhibition in dendrites. Neuron 75: 330–341. 22841317 10.1016/j.neuron.2012.05.015

[32] Araya R, Jiang J, Eisenthal KB, Yuste R (2006) The spine neck filters membrane potentials. Proceedings of the National Academy of Sciences 103: 17961–17966. 10.1073/pnas.0608755103 PMC1693855 17093040

[33] Yuste R (2011) Dendritic spines and distributed circuits. Neuron 71: 772–781. 21903072 10.1016/j.neuron.2011.07.024 PMC4071954

[34] Magee JC, Cook EP (2000) Somatic epsp amplitude is independent of synapse location in hippocampal pyramidal neurons. Nature neuroscience 3: 895–903. 10966620 10.1038/78800

[35] Harnett MT, Makara JK, Spruston N, Kath WL, Magee JC (2012) Synaptic amplification by dendritic spines enhances input cooperativity. Nature 491: 599–602. 23103868 10.1038/nature11554 PMC3504647

[36] Gasparini S, Migliore M, Magee JC (2004) On the initiation and propagation of dendritic spikes in ca1 pyramidal neurons. The Journal of neuroscience 24: 11046–11056. 15590921 10.1523/JNEUROSCI.2520-04.2004 PMC6730267

[37] Sun Y, Zhou D, Rangan AV, Cai D (2009) Library-based numerical reduction of the hodgkin–huxley neuron for network simulation. Journal of computational neuroscience 27: 369–390. 19401809 10.1007/s10827-009-0151-9

[38] Dayan P, Abbott L (2001) Theoretical neuroscience: Computational and mathematical modeling of neural systems. Cambridge: MIT Press.

[39] Crank J, Nicolson P (1947) A practical method for numerical evaluation of solutions of partial differential equations of the heat-conduction type. In: Mathematical Proceedings of the Cambridge Philosophical Society. Cambridge Univ Press, volume 43, pp. 50–67.

[40] Cannon R, Turner D, Pyapali G, Wheal H (1998) An on-line archive of reconstructed hippocampal neurons. Journal of neuroscience methods 84: 49–54. 9821633 10.1016/s0165-0270(98)00091-0

[41] Destexhe A, Mainen ZF, Sejnowski TJ (1994) An efficient method for computing synaptic conductances based on a kinetic model of receptor binding. Neural computation 6: 14–18.

[42] Destexhe A, Mainen ZF, Sejnowski TJ (1994) Synthesis of models for excitable membranes, synaptic transmission and neuromodulation using a common kinetic formalism. Journal of computational neuroscience 1: 195–230. 8792231 10.1007/BF00961734

[43] Stuart G, Spruston N (1998) Determinants of voltage attenuation in neocortical pyramidal neuron dendrites. The Journal of neuroscience 18: 3501–3510. 9570781 10.1523/JNEUROSCI.18-10-03501.1998 PMC6793161

[44] Magee JC, Johnston D (1995) Characterization of single voltage-gated na+ and ca2+ channels in apical dendrites of rat ca1 pyramidal neurons. The Journal of Physiology 487: 67–90. 7473260 10.1113/jphysiol.1995.sp020862 PMC1156600

[45] Hoffman DA, Magee JC, Colbert CM, Johnston D (1997) K+ channel regulation of signal propagation in dendrites of hippocampal pyramidal neurons. Nature 387: 869–875. 9202119 10.1038/43119

[46] Migliore M, Hoffman D, Magee J, Johnston D (1999) Role of an a-type k+ conductance in the back-propagation of action potentials in the dendrites of hippocampal pyramidal neurons. Journal of computational neuroscience 7: 5–15. 10481998 10.1023/a:1008906225285

[47] Magee JC (1998) Dendritic hyperpolarization-activated currents modify the integrative properties of hippocampal ca1 pyramidal neurons. The Journal of neuroscience 18: 7613–7624. 9742133 10.1523/JNEUROSCI.18-19-07613.1998 PMC6793032

[48] Andrásfalvy BK, Magee JC (2001) Distance-dependent increase in ampa receptor number in the dendrites of adult hippocampal ca1 pyramidal neurons. The Journal of Neuroscience 21: 9151–9159. 11717348 10.1523/JNEUROSCI.21-23-09151.2001 PMC6763889

[49] Smith MA, Ellis-Davies GC, Magee JC (2003) Mechanism of the distance-dependent scaling of schaffer collateral synapses in rat ca1 pyramidal neurons. The Journal of physiology 548: 245–258. 12598591 10.1113/jphysiol.2002.036376 PMC2342790

[50] Nicholson DA, Trana R, Katz Y, Kath WL, Spruston N, et al (2006) Distance-dependent differences in synapse number and ampa receptor expression in hippocampal ca1 pyramidal neurons. Neuron 50: 431–442. 16675397 10.1016/j.neuron.2006.03.022

[51] Carnevale N, Hines M (2006) The NEURON book. Cambridge: Cambridge Univ. Press.

